# One-year treatment with mometasone furoate in chronic obstructive pulmonary disease

**DOI:** 10.1186/1465-9921-9-73

**Published:** 2008-11-13

**Authors:** Peter MA Calverley, Stephen Rennard, Harold S Nelson, Jill P Karpel, Eduardo H Abbate, Paul Stryszak, Heribert Staudinger

**Affiliations:** 1 Department of Medicine, University Hospital Aintree, Liverpool, UK; 2 University of Nebraska Medical Center, Omaha, NE, USA; 3 Department of Medicine, National Jewish Medical and Research Center, Denver, CO, USA; 4 North Shore University Hospital, New Hyde Park, NY, USA; 5 Asociacion Argentina de Medicina Respiratoria, Buenos Aires, Argentina; 6 Schering-Plough Research Institute, Kenilworth, NJ, USA

## Abstract

Many patients with chronic obstructive pulmonary disease (COPD) are treated with twice daily (BID) inhaled corticosteroids (ICS). This study evaluated whether daily PM mometasone furoate administered via a dry powder inhaler (MF-DPI) was equally effective compared to twice daily dosing.

In a 52-week, randomized, double-blind, placebo-controlled study, 911 subjects with moderate-to-severe COPD managed without ICS received MF-DPI 800 μg QD PM, MF-DPI 400 μg BID, or placebo. The change from baseline in postbronchodilator forced expiratory volume in 1 second (FEV_1_), total COPD symptom scores, and health status as well as the percentage of subjects with a COPD exacerbation were assessed. Adverse events were recorded.

Mometasone furoate administered via a dry powder inhaler 800 μg QD PM and 400 μg BID significantly increased postbronchodilator FEV_1 _from baseline (50 mL and 53 mL, respectively, versus a 19 mL decrease for placebo; *P *< 0.001). The percentage of subjects exacerbating was significantly lower in the pooled MF-DPI groups than in the placebo group (*P *= 0.043). Subjects receiving MF-DPI 400 μg BID reported a statistically significant (19%) reduction in COPD symptom scores compared with placebo (*P *< 0.001). Health status as measured with St. George's Respiratory Questionnaire (SGRQ) improved significantly in all domains (Total, Activity, Impacts, and Symptoms) in the pooled MF-DPI groups versus placebo (*P *≤ 0.031). MF-DPI treatment was well tolerated.

Once-daily MF-DPI improved lung function and health status in subjects with moderate-to-severe COPD and was comparable to BID MF-DPI.

## Background

Chronic obstructive pulmonary disease (COPD), now recognized as a major chronic disease, is associated with significant mortality, morbidity, and healthcare expense [1,2]. The underlying pathology of COPD in both the airways and the alveoli is inflammatory in nature[3], with the inflammation increasing as the disease progresses. Important differences between asthma and COPD pathology influence the response to treatment in each disease [4]. In asthma, inhaled corticosteroids (ICS) reduce airway inflammation and improve lung function, as well as a range of clinical endpoints [5]. In COPD, ICS treatments have only a minimal effect on airway pathology [6], which may explain their limited effect on rate of decline of lung function [7]. However, the change in spirometric deterioration is only one of many important outcomes in COPD.

Among the major goals of therapy in COPD is the reduction of exacerbations, control of symptoms, and slowing the decline in health status, which may be distinct from lost lung function. A number of large randomized controlled trials have shown that treatment with ICS can achieve these effects [8-11]. In addition, discontinuation of ICS therapy in subjects also using ipratropium rapidly led to the onset of recurrent exacerbations in many subjects [12]. Based on this evidence, the Global Initiative for Chronic Obstructive Lung Disease (GOLD) [13,14] recommends the use of ICS in severe COPD with forced expiratory volume in 1 second (FEV_1_) < 50% predicted and repeated exacerbations that require treatment with antibiotics or oral corticosteroids, a view supported by other evidence-based recommendations [15].

Mometasone furoate (MF), a synthetic 17-heterocyclic corticosteroid used for more than 15 years in the management of nasal inflammation and dermatoses, is now licensed for the treatment of bronchial asthma. MF appears to have a favorable side-effect profile and offers the practical advantage of a long duration of action, which permits QD therapy [16-19]. Treatment with MF administered via a dry powder inhaler (MF-DPI) 400 μg QD or, in many patients, 200 μg QD PM, is effective and well tolerated in mild and moderate persistent asthma [16-19]. MF-DPI administered at a total daily dose of 800 μg/day (400 μg BID) is effective in patients with severe asthma previously dependent on maintenance oral corticosteroid therapy [20]. However, its effect, if any, in patients with COPD has not been established.

We hypothesized that MF-DPI 800 μg once daily in the evening would be statistically superior to placebo for changes from baseline in postbronchodilator FEV_1 _and total COPD symptoms, or the percentage of subjects with one or more exacerbations, or both. To test this hypothesis, a randomized, double blind, parallel-group, placebo-controlled trial was conducted comparing the efficacy and safety of MF-DPI 800 μg once daily in the evening and MF-DPI 400 μg twice daily with placebo and with each other in subjects with COPD managed without ICS.

## Methods

### Study subjects

All subjects provided written informed consent approved by an Independent Ethics Committee or Institutional Review Board. All subjects had a diagnosis of COPD based on currently accepted criteria [14], and were current smokers who failed a mandatory smoking cessation program or self-reported ex-smokers who had stopped smoking ≥ 12 months before the study. Eligible subjects had a prebronchodilator FEV_1_/FVC (forced vital capacity) ratio ≤ 70%, postbronchodilator FEV_1 _between 30% and 70% predicted, and low postbronchodilator FEV_1 _reversibility (< 10% of predicted normal). Per protocol, subjects did not receive inhaled, oral, or parenteral corticosteroids for 6 weeks prior to screening. During the study, ipratropium bromide, theophylline, short- and long-acting β_2_-adrenergic agonists (with appropriate washout before study visits) were allowed.

Subjects with a clinical history of asthma or any other clinically significant medical illness other than COPD were excluded. Other exclusion criteria included a COPD exacerbation within 3 months before the baseline visit; ventilator support for respiratory failure within the past year; lobectomy, pneumonectomy, or lung volume reduction surgery; lung cancer within the past 5 years; nasal continuous positive airway pressure or oxygen use > 2 L/min or for >2 hours per day; initiation of pulmonary rehabilitation within the past 3 months; treatment with chronic or prophylactic antibiotics; inability to use the MF-DPI inhaler; and < 80% adherence in recording diary data between screening and baseline.

### Study design

This was a randomized double-blind, placebo-controlled, parallel-group study in males and females of any race, ≥ 40 years of age, with a clinical history and spirometry diagnostic of COPD. The study was conducted at 95 sites in 11 countries. Subjects underwent a 2-week run-in before randomization, in which spirometry results, exacerbations, symptom scores, and health status were recorded to ensure clinical stability. Subjects who had an exacerbation during the run-in were rescreened 2 or 6 weeks after completion of antibiotic or oral corticosteroid therapy, respectively. These measurements were made at each subsequent clinic visit (weeks 1, 4, 13, 26, 39, and 52). Telephone contacts occurred at weeks 8, 17, 21, 30, 34, 43, and 47 to reinforce adherence to study procedures and monitor adverse events, exacerbations, and concomitant medication use. Eligible subjects were randomized via computer-generated code in a ratio of 2:2:1:1 to 52 weeks of treatment with MF-DPI 800 μg QD PM, MF-DPI 400 μg BID, placebo QD PM, or placebo BID. Dosing regimens (QD or BID) were not blinded. Spirometry was performed before, and 30 minutes after, inhalation of albuterol 400 μg. Testing was conducted to American Thoracic Society standards [21], and the reference values of Crapo et al [22] were used to determine the % predicted FEV_1_. Subjects maintained twice-daily diaries documenting symptom scores, daily use of rescue medication, and cigarette consumption. Exacerbations requiring treatment with antibiotics or oral corticosteroids were recorded throughout the study. Subjects who discontinued during the treatment period continued to maintain their diaries, which were reviewed at follow-up visits. Health-related quality of life was evaluated at baseline and every 3 months.

### Analysis

The primary efficacy variable was the change from baseline in postbronchodilator FEV_1_. Other prespecified efficacy variables were the percentage of subjects with 1 or more exacerbations during the study and the change from baseline in total COPD symptom scores. An exacerbation was defined as a clinically significant worsening of COPD symptoms requiring treatment with antibiotics and/or systemic steroids. Subjects who experienced 3 COPD exacerbations or needed more than 3 weeks of treatment for an exacerbation were discontinued. Total symptom scores were the average of daytime and nighttime scores for difficulty breathing, coughing, and wheezing. Subjects recorded scores for each of these symptoms in daily diaries. Difficulty breathing was rated on separate scales for daytime and nighttime scores of 0 (none) to 4 (severe), while coughing and wheezing were rated on a scale of 0 (none) to 3 (very uncomfortable). Secondary efficacy evaluations were changes from baseline in St. George's Respiratory Questionnaire (SGRQ) and 36-item Short Form (SF-36) scores, prebronchodilator FEV_1_, prebronchodilator and postbronchodilator FVC and forced expiratory flow between 25% and 75% of vital capacity (FEF_25%–75%_), and individual daytime and nighttime symptom scores.

Safety assessments included monitoring of adverse events, with specific oropharyngeal and forearm examinations, and assessment of vital signs at all study visits. Physical examinations and laboratory tests were done at screening and the final visit. Plasma cortisol levels were assessed at the baseline and final visits in subjects at approximately 15 centers; samples were taken at 4 AM, 5 AM, 6 AM, 7 AM, 8 AM, 9 AM, 10 AM, 12 PM, 4 PM, 8 PM, and 11 PM. In other selected centers, bone mineral density (BMD) in the lumbar spine and proximal femur, using dual-energy X-ray absorptiometry (DXA) was assessed. The bone scans were performed by local radiologists and the results were reviewed by Synarc, Inc. (Portland, OR).

The efficacy and safety analyses were based on all randomized subjects (intent-to-treat population). A confirmatory analysis of subjects who met key eligibility and evaluability criteria was also performed. Results are expressed as least squares means (± SD). A longitudinal analysis-random coefficient model was used to evaluate treatment effects on postbronchodilator FEV_1 _and COPD symptom scores. Longitudinal analysis of results for postbronchodilator FEV_1 _extracted sources of variability due to smoking status, treatment, number of days on treatment, and treatment-by-time interaction, with a random slope and intercept for each subject. An unstructured covariance matrix, with variance of random intercepts and slopes and covariance between intercepts and slopes, was chosen to allow full flexibility of the model. The Cochran-Mantel-Haenzsel test was used to analyze exacerbation frequency.

The primary hypothesis was that 800 μg QD PM would be superior to placebo with respect to changes from baseline in postbronchodilator FEV_1 _and either total COPD symptom scores or proportion of subjects with one or more exacerbations during the study. The study design required that 780 subjects meet the criteria for evaluation of the 3 prespecified efficacy variables. To control for type I error, the pooled MF-DPI groups were to be compared with the pooled placebo groups. If pooled MF-DPI was significantly superior to pooled placebo for FEV_1 _and at least 1 of the other prespecified criteria, then the following comparisons were made: MF-DPI 800 μg QD PM versus pooled placebo, MF-DPI 400 μg BID versus placebo. If at least 1 of the MF-DPI treatments was superior to placebo, then the 2 MF-DPI treatments were compared with each other. Mean changes from baseline were compared at a 2-sided α = 0.05, providing at least 90% power to detect between the treatment means a difference of 50 mL in postbronchodilator FEV_1_, a difference of 0.2 in total symptom scores, and a 16% difference in the proportion of subjects having at least 1 exacerbation. This power was maintained throughout the stepwise comparisons of MF-DPI treatments with placebo. A post hoc analysis was performed to test whether the MF-DPI 800 μg QD PM and 400 μg BID were equivalent in terms of their effects on the co-primary endpoints of FEV_1_, total symptom scores, and exacerbations. The equivalence margin chosen was 50% of the difference between placebo and MF-DPI 400 μg BID. The post hoc analysis had 78% power to detect equivalence with regard to FEV_1_, 59% power to detect equivalence with regard to total symptom scores, and 21% power to detect equivalence with regard to exacerbations.

To protect against inflation of the error rate for multiple primary endpoints, comparisons of exacerbations and symptom scores were adjusted using a modified Bonferroni correction, with a 2-sided α = 0.025 for the more significant comparisons and α = 0.05 for the less significant comparisons. This was determined by the size of the results from the symptom score and exacerbation comparisons, the smaller of which was considered more significant as it required α = 0.025 for statistical significance, and the results of the larger value considered less significant, as it required α = 0.05 for statistical significance.

## Results

**Figure 1:**
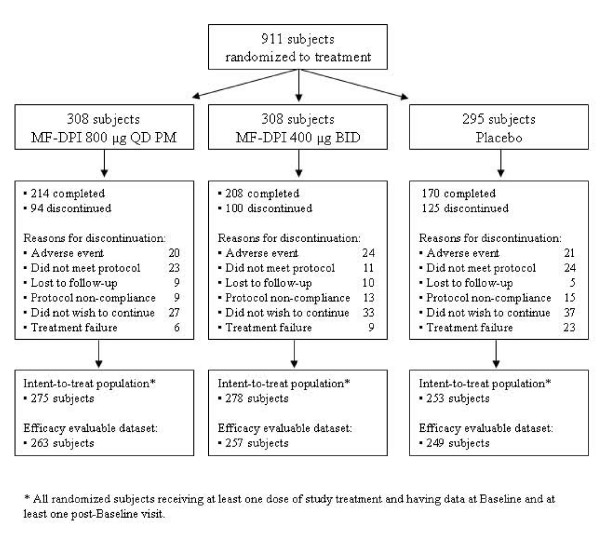
Disposition of study subjects.

A total of 911 subjects were randomized to treatment with MF-DPI 800 μg QD PM (n = 308), MF-DPI 400 μg BID (n = 308), or placebo (n = 295). Of these, 319 subjects discontinued treatment (Figure 1). The proportion of subjects discontinuing because of treatment failure was higher in the placebo group (8%) than in the MF-DPI groups (2%). The time to discontinuation was longest in the MF-DPI 800 μg QD PM group and shortest in the placebo group, with greater separation between active treatments and placebo over the treatment period. All treatment groups were similar with regard to baseline demographics and disease characteristics (Table 1), smoking status, previous ICS use, and concomitant long-acting β_2_-agonist use. Of the randomized subjects, 250 had entered the prescreening smoking cessation program. One hundred eleven (44%) of these 250 subjects had completed the program and 139 (56%) had discontinued the program. At baseline, approximately 30 subjects in each treatment group (n = 92) had DXA scans of the lumbar spine and femoral neck; approximately 20 in each group (n = 65) had DXA scans at study endpoint. These subjects had comparable demographic and disease characteristics to each other and to the overall study population. Their mean age was 65 years, 38% were females and 62% were males, and their mean postbronchodilator FEV_1 _was 1.46 L.

**Table 1:** Baseline demographics and disease characteristics in all randomized subjects

	**MF-DPI 800 μg QD PM (n = 308)**	**MF-DPI 400 μg BID (n = 308)**	**Placebo (n = 295)**
Mean age, y	65.3	65.0	65.0
Sex, n (%)			
Women	95 (31)	102 (33)	92 (31)
Men	213 (69)	206 (67)	203 (69)
Race, n (%)			
White	271 (88)	264 (86)	252 (85)
Non-white	37 (12)	44 (14)	43 (15)
Mean body mass index, kg/m^2^	26.7*	26.1*	27.1
Mean COPD duration, y	7.33*	7.31^†^	7.26
Pulmonary function			
Prebronchodilator FEV_1_, L	1.32	1.25	1.26
Postbronchodilator FEV_1_, L	1.45	1.38	1.41
% FEV_1 _predicted			
Prebronchodilator	43	42	42
Postbronchodilator	47	46	47
Reversibility (%)	4	4	5
COPD severity, n (%)^‡^			
FEV_1 _50%–<80% predicted	97 (32)	88 (29)	81 (28)
FEV_1 _30%–<50% predicted	142 (46)	136 (44)	127 (43)
FEV_1 _< 30% predicted	60 (20)	67 (22)	67 (23)
Missing^§^	8 (3)	17 (6)	20 (7)

BID = twice daily; COPD = chronic obstructive pulmonary disease; FEV_1 _= forced expiratory volume in 1 second; MF-DPI = mometasone furoate administered via a dry powder inhaler.

*n = 305

^†^n = 306

^‡^Sum of % values may not be 100% due to rounding.

^§^Screen failures or inadequate data (at least one valid measure of prebronchodilator or postbronchodilator FEV_1_, but not both, were available).

### Pulmonary function

**Figure 2:**
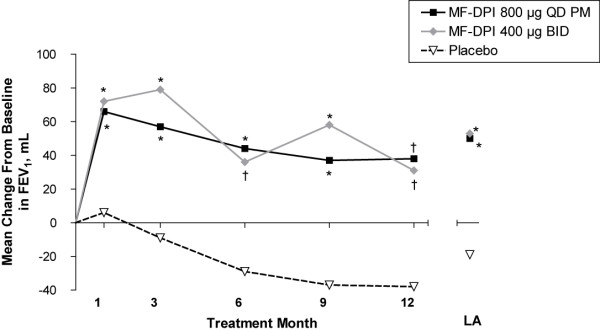
**Changes from baseline in postbronchodilator FEV_1_**. BID = twice daily; FEV_1 _= forced expiratory volume in 1 second; LA = longitudinal average; MF-DPI = mometasone furoate delivered via a dry powder inhaler; QD PM = once-daily in the evening. *P ≤ 0.001 vs placebo; ^†^*P *≤ 0.006 vs placebo.

Significant improvements in postbronchodilator FEV_1 _were observed in both MF-DPI groups over the 1-year treatment period (Table 2 and Figure 2). In addition, both MF-DPI regimens were superior to placebo (*P *≤ 0.012) for changes from baseline in prebronchodilator FEV_1 _and prebronchodilator and postbronchodilator FVC and FEF_25%–75% _(Table 2). The MF-DPI treatments were consistently superior to placebo in LABA users and nonusers. No significant differences were observed between MF-DPI treatments in any of the pulmonary function variables, and there were no differences in treatment effect based on subjects' sex, age, prebronchodilator FEV_1_% predicted, or prior medical history. However, the response to MF-DPI treatment was greater in ex-smokers (those who quit smoking ≥ 10 months before baseline) than in those who continued smoking. Mean changes in cigarette use during treatment were low (< 2 cigarettes/day) in all treatment groups. In ex-smokers, postbronchodilator FEV_1 _increased approximately 50 mL with MF-DPI compared with a decrease (-11 mL) with placebo. In current smokers, postbronchodilator FEV_1 _increased (29 mL) in the MF-DPI 800 μg QD PM group, but decreased in the MF-DPI 400 μg BID and placebo groups (-9 mL and -41 mL, respectively).

**Table 2:** Changes from baseline in pulmonary function*

	**MF-DPI 800 μg QD PM 800(n = 275)**	**MF-DPI 400 μg BID (n = 278)**	**Placebo (n = 256)**
Prebronchodilator FEV_1_, L (pSD)			
Longitudinal average	0.029 (0.182)^†^	0.041 (0.182)^†^	-0.034 (0.182)
ΔMF-DPI – placebo (95% CI)	0.063 (0.033–0.094)	0.075 (0.044–0.105)	
Postbronchodilator FEV_1_, L (SD)			
Longitudinal average	0.050 (0.182)^†^	0.053 (0.183)^†^	-0.019 (0.176)
ΔMF-DPI – placebo (95% CI)	0.069 (0.040–0.098)	0.072 (0.044–0.102)	
Prebronchodilator FEF_25%–75%_, L/se(pSD)			
Longitudinal average	0.027 (0.169)^‡^	0.037 (0.169)^†^	-0.016 (0.169)
ΔMF-DPI – placebo (95% CI)	0.043 (0.014–0.071)	0.053 (0.024–0.082)	
Postbronchodilator FEF_25%–75%_, L/s (pSD)			
Longitudinal average	0.049 (0.185)^‡^	0.042 (0.185)^‡^	0.02 (0.185)
ΔMF-DPI – placebo (95% CI)	0.047 (0.015–0.079)	0.040 (0.09–0.072)	
Prebronchodilator FVC, L (pSD)			
Longitudinal average	0.031 (0.328)^†^	0.045 (0.328)^†^	-0.066 (0.328)
ΔMF-DPI – placebo (95% CI)	0.09 (0.042–0.153)	0.111 (0.056–0.166)	
Postbronchodilator FVC, L (pSD)			
Longitudinal average	0.066 (0.316)^†^	0.044 (0.316)^‡^	-0.028 (0.316)
ΔMF-DPI – placebo (95% CI)	0.094 (0.040–0.148)	0.072 (0.018–0.125)	

BID = twice daily; FEF_25%–75% _= forced expiratory flow between 25% and 75% of vital capacity; FEV_1 _= forced expiratory volume in 1 second; FVC = forced vital capacity; MF-DPI = mometasone furoate administered via a dry powder inhaler; pSD = pooled standard deviation.

*This table shows spirometry results in subjects for whom both pre- and postbronchodilator data were available.

^†^*P *< 0.001 vs placebo

^‡^*P *≤ 0.009 vs placebo

### Exacerbations

A total of 334 randomized subjects (37%) had 1 or more COPD exacerbations during treatment: 105 (34%) in the MF-DPI 800 μg QD PM group, 107 (35%) in the MF-DPI 400 μg BID group, and 122 (41%) in the placebo group. The difference between the pooled MF-DPI groups and placebo was significant (*P *= 0.043). Analysis by the log-rank test showed that each MF-DPI treatment significantly (*P *< 0.019) prolonged the time to first exacerbation compared with placebo. In each MF-DPI group, 46% of those with a history of ≥ 3 exacerbations exacerbated during the treatment period versus 30% of those with a history < 3 exacerbations. In the placebo group, 61% of those with a history of ≥ 3 exacerbations exacerbated during the treatment period, whereas 34% of patients with < 3 previous exacerbations exacerbated. Treatment efficacy with respect to second and third exacerbations was evaluated as the total number of events divided by the total follow-up time. With this measurement the exacerbation rates for MF-DPI 800 μg QD PM, MF-DPI 400 μg BID, and placebo were 0.62, 0.65, and 0.96, respectively.

A greater proportion of subjects in the placebo group had severe exacerbations (resulting in hospitalization, use of both oral steroids and antibiotics, or additional oral steroids) than did those in either of the MF-DPI groups. A greater proportion of exacerbations in the MF-DPI groups were treated with antibiotics alone. These differences were not statistically significant (Table 3). Also, subjects with baseline FEV_1 _< 50% predicted (ie, GOLD Stages III-IV) had more exacerbations than those with FEV_1 _> 50% predicted (ie, GOLD Stages I-II). For subjects in GOLD Stages I-II, exacerbations were reported for 18% in the MF-DPI 800 μg QD PM group, 27% in the MF-DPI 400 μg BID group, and 35% in the placebo group. For subjects in GOLD Stages III-IV, exacerbations were reported for 43% in the MF-DPI 800 μg QD PM group, 41% in the MF-DPI 400 μg BID group, and 48% in the placebo group.

**Table 3:** Exacerbations classified by severity

	**MF-DPI 800 μg QD PM**	**MF-DPI 400 μg BID**	**Placebo**
Total number of exacerbations	154	159	207
Hospitalizations, n (%)	12 (8)	12 (8)	20 (10)
Use of both oral steroid and antibiotic, n (%)	50 (32)	55 (35)	75 (36)
Use of oral steroid alone, n (%)	26 (17)	31 (20)	51 (25)
Use of antibiotic alone, n (%)	66 (43)	61 (38)	61 (30)
Exacerbation rates*			
More severe exacerbations^†^	0.36^‡^	0.41^§^	0.69
All exacerbations	0.62^§^	0.65^§^	0.96

BID = twice daily; MF-DPI = mometasone furoate administered via a dry powder inhaler; QD PM = once daily in the evening.

*Ratio between total number of events and total duration of treatment across all subjects.

^†^Resulting in hospitalization, use of both oral steroids and antibiotics, or of oral steroids alone, as opposed to use of antibiotics alone.

^‡^*P *= 0.002 vs placebo

^§^*P *≤ 0.022 vs placebo

Of the 258 patients who currently smoked, 93 exacerbated 1 or more times during the study, with 28 (32%) in the MF-DPI 800 μg QD PM group, 34 (36%) in the MF-DPI 400 μg BID group, and 31 (40%) in the placebo group. Of the 653 ex-smokers, 241 subjects exacerbated 1 or more times, with 77 (35%) in the MF-DPI 800 μg QD PM group, 73 (34%) in the MF-DPI 400 μg BID group, and 91 (42%) in the placebo group.

### Symptom scores

Total COPD symptom scores (average of daytime and nighttime scores), improved significantly (*P *< 0.05) from baseline over the 12-month treatment period with both MF-DPI 400 μg BID (-0.53) and MF-DPI 800 μg QD PM (-0.34) compared with placebo (-0.12). A confirmatory analysis based on the efficacy evaluable data set and all randomized subjects while on treatment indicated a significant improvement from baseline in total COPD symptom scores for each active treatment group compared with placebo (*P *≤ 0.021). For individual symptom scores (Table 4), significant improvements (*P *< 0.025) in daytime difficulty breathing scores were observed for each active treatment compared with placebo and for most other scores for the MF-DPI 400 μg BID group.

**Table 4:** Changes from baseline in COPD symptom scores*

	**MF-DPI 800 μg QD PM (n = 282)**	**MF-DPI 400 μg BID (n = 283)**	**Placebo (n = 263)**
**Total COPD symptom scores (daytime)**			
Baseline	2.66	2.78	2.64
Longitudinal average	-0.36	-0.57^†^	-0.11
**Total COPD symptom scores (nighttime)**			
Baseline	2.54	2.73	2.65
Longitudinal average	-0.30	-0.50^†^	-0.12
**Daytime symptom scores**			
*Difficulty Breathing*			
Baseline	1.06	1.14	0.99
Longitudinal average	-0.11^‡^	-0.23^†^	0.02
*Coughing*			
Baseline	0.92	0.91	0.97
Longitudinal average	-0.14	-0.16	-0.10
*Wheezing*			
Baseline	0.70	0.74	0.68
Longitudinal average	-0.12	-0.18^†^	-0.04
**Nighttime Symptom Scores**			
*Difficulty breathing*			
Baseline	1.00	1.10	1.01
Longitudinal average	-0.10	-0.18^†^	0.01
*Coughing*			
Baseline	0.90	0.91	0.96
Longitudinal average	-0.12	-0.16	-0.08
*Wheezing*			
Baseline	0.66	0.73	0.69
Longitudinal average	-0.08	-0.17^†^	-0.05

BID = twice daily; COPD = chronic obstructive pulmonary disease; MF-DPI = mometasone furoate administered via a dry powder inhaler.

A *P *value of 0.025 was the upper limit of statistical significance based on the modified Bonferroni correction used in this analysis.

*This table shows symptom-score results for subjects in whom baseline and post-baseline diary data were available.

^†^*P *≤ 0.003 vs placebo

^‡^*P *< 0.025 vs placebo

### Health status

Scores for the SGRQ Total, Activity, Impacts, and Symptoms domains (Table 5) improved significantly in the pooled MF-DPI groups compared with the pooled placebo groups (*P *≤ 0.031). A significant improvement in the SF-36 physical component summary score was also observed in the pooled MF-DPI groups compared with placebo (*P *< 0.05). For the individual groups, treatment with MF-DPI 800 μg QD PM significantly improved SGRQ Symptoms scores (-5.77, *P *= 0.050). Compared with placebo, treatment with MF-DPI 400 μg BID significantly improved SGRQ Total scores (-3.99, *P *= 0.008), Impacts scores (-3.41, *P *= 0.042), and Symptoms scores (-6.85, *P *= 0.009).

**Table 5:** Changes from baseline in SGRQ and SF-36 scores*

	**MF-DPI**^†^		**Placebo**^†^		**MF-DPI – Placebo**	
	**n**	**Mean**	**n**	**Mean**	**Mean**	***P *value**

SGRQ scores						
Total	504	-3.92	241	-0.64	-3.28	0.003
Activity	506	-4.20	242	-1.05	-3.15	0.021
Impacts	516	-2.81	244	-0.25	-2.58	0.031
Symptoms	534	-7.21	248	-2.26	-4.95	0.004
SF-36 PCS score	503	1.05	235	0.01	1.04	0.050
SF-36 MCS score	503	0.25	235	-0.32	0.57	0.372

MCS = mental component summary; MF-DPI = mometasone furoate administered via a dry powder inhaler; PCS = physical component summary; SF-36 = 36-item short form; SGRQ = St. George's respiratory questionnaire.

*This table shows results for subjects who completed HRQOL questionnaires at both baseline and endpoint.

^†^Pooled treatment groups.

### Safety

The incidence rates of any treatment-emergent adverse event were comparable in the 3 treatment groups (Table 6). Treatment-related adverse events were reported by 27% of subjects taking MF-DPI 800 μg QD PM, 28% of subjects taking MF-DPI 400 μg BID, and 20% of placebo-treated subjects. Most adverse events reported were mild to moderate in severity; no life-threatening events were considered to be related to study medication. Oral candidiasis was the most frequent treatment-related adverse event; 10%–11% for MF and 3% for placebo. The incidence of bruising was 14% in each MF-DPI group and 11% in the placebo group. In both groups, the incidence rates of fracture, osteoporosis, and cataracts were ≤ 1%.

**Table 6:** Adverse events in all randomized subjects

	**MF-DPI 800 μg QD PM (n = 308)**	**MF-DPI 400 μg BID (n = 308)**	**Placebo (n = 295)**
Any adverse event, n (%)	224 (73)	228 (74)	204 (69)
URTI, n (%)	82 (27)	82 (27)	71 (24)
Oral candidiasis, n (%)	34 (11)	30 (10)	10 (3)
Pharyngitis, n (%)	26 (8)	28 (9)	24 (8)
Bruise/bruising*, n (%)	46 (15)	45 (14)	33 (11)
New forearm bruising^†^, n (%)	27 (10)	27 (10)	21 (7)
Back pain, n (%)	23 (7)	15 (5)	10 (3)
COPD aggravated, n (%)	11 (4)	12 (4)	14 (5)
Pneumonia, n (%)	12 (4)	13 (4)	6 (2)

BID = twice daily; COPD = chronic obstructive pulmonary disease; MF-DPI = mometasone furoate administered via a dry powder inhaler; URTI = upper respiratory tract infection.

*Either adverse event, which have different reporting codes, could be reported.

†Visual forearm inspection completed at each visit. New forearm bruises = 5 cm in diameter were captured in a separate module of the case report form. All new bruises, regardless of size and location, were captured in the adverse event module.

Ten subjects died during treatment or within 30 days of the last dose of study treatment: 2 in the MF-DPI 800 μg QD PM group, 5 in the MF-DPI 400 μg BID group, and 3 in the placebo group. None of the deaths was due to respiratory disease or lung cancer or considered to be related to treatment. Serious adverse events were reported by 142 subjects during the treatment period: 44 (14%) in the MF-DPI 800 μg QD PM group, 47 (15%) in the MF-DPI 400 μg BID group, and 51 (17%) in the placebo group. The only serious events reported by > 1% of any treatment group were pneumonia (2% in each MF-DPI group and 1% in the placebo group) and COPD aggravated (4% in each MF-DPI group and 5% in the placebo group).

The total lumbar spine BMD increased slightly (0.857%) at endpoint in the MF-DPI 800 μg QD PM group and decreased slightly in the MF-DPI 400 μg BID and placebo groups (-0.944% and -0.068%, respectively). Likewise, total femoral BMD increased slightly (0.347%) at endpoint in the MF-DPI 800 μg QD PM group and decreased slightly in the MF-DPI 400 μg BID and placebo groups (-2.002% and -0.677%, respectively). Femoral neck results were similar in direction and inference between treatment groups to the total femoral BMD results. No significant differences were observed between any of the treatment groups.

At endpoint, there was a significant decrease of 22.9% in the plasma cortisol level for subjects in the MF-DPI 400 μg BID treatment group compared with an increase of 3.7% for subjects in the MF-DPI 800 μg QD PM group (*P *= 0.040) and 5.3% for subjects in the placebo group (*P *= 0.007).

## Discussion

This is the first randomized controlled trial to report the effects of once-daily therapy with an ICS in subjects with COPD. MF-DPI produced improvements in a range of efficacy endpoints that were generally comparable in magnitude to those reported with other ICSs when studied in subjects with moderate-to-severe COPD [23,24] This study also demonstrated that MF-DPI 800 μg QD PM has comparable efficacy to dividing the dose into a BID regimen, especially for number and severity of exacerbations.

The primary outcome of this study was the change in postbronchodilator FEV_1_, which has been noted to improve in some, but not all, previous trials of ICS in COPD [8,9,23,25,26]. With both MF-DPI dosing regimens, there was an early and sustained improvement in FEV_1_, which was comparable to that seen with fluticasone 500 μg BID in similar subjects [9,23,26]. In subjects receiving placebo, postbronchodilator FEV_1 _declined during the study, whereas it improved with both MF-DPI regimens. This change occurred independently of the initial degree of airflow obstruction, but was influenced by the patient's smoking status. Ex-smokers showed a greater improvement in postbronchodilator FEV_1 _with the ICS than subjects who continued to smoke despite a smoking cessation program. This effect was not explained by the previously described short-term improvement in lung function that occurs in individuals who stop smoking, a finding which is more evident when lung function is better preserved[27]. Subjects who successfully quit smoking during the smoking cessation program were ineligible for the study because they would show improvements in lung function that would interfere with the evaluation of treatment effects. The differences in FEV_1 _between the active treatment and placebo groups were similar to the differences in the acute response of FEV_1 _to high doses of oral prednisolone given for a shorter period [28]. The relative lack of response among continuing smokers resembles the recently described clinical corticosteroid resistance in subjects with bronchial asthma who continued to smoke [29]. A molecular basis for this form of relative corticosteroid resistance has been proposed and may be relevant to the pathogenesis of COPD[30].

The number of exacerbations reported by subjects in the present trial was lower than that reported in some previous studies, despite the many participants who had severe COPD, which is commonly associated with more frequent exacerbations [31]. Unlike the ISOLDE and TORCH studies that accrued exacerbations over 3 years, subjects were studied for 12 months and a history of either chronic bronchitis or previous exacerbations was not an enrollment criterion [23,32]. The use of regular telephone contacts may have improved patient compliance with therapy and enhanced the benefit of participating in a clinical trial [33]. Although the number of events was lower than anticipated, the time to the first exacerbation was significantly increased in subjects receiving ICS. This form of analysis is a statistically efficient way of testing for an effect on exacerbation rate and is most appropriate when the subjects drop out in significant numbers during the trial. This was the case here, with an excessive number of subjects randomized to placebo withdrawing. This finding has been seen in other clinical trials using ICS [23,24,28,32,33], and subjects who drop out in this way are normally those who are deteriorating most rapidly in terms of lung function and health status [33]. The ability of subjects randomized to MF-DPI to complete the study is a further indication of a positive treatment effect. Furthermore, more placebo-treated subjects had severe exacerbations that required hospitalization or additional treatment, other than antibiotic therapy, suggesting that MF-DPI reduces both the number and severity of exacerbations. Based on pooled results for the MF-DPI groups, the number needed to treat to prevent an exacerbation in one year was 14.

The exacerbation rate is an important determinant of health status [34]. Despite the relatively low number of events observed, a significant improvement was observed in the SGRQ Total score in subjects receiving MF-DPI during the study. This was similar to the annual difference in SGRQ between placebo and active treatment in the ISOLDE study [11] and was comparable in magnitude to the improvement in health status in other studies where ICS treatments were withdrawn at random and subjects followed subsequently [12,26,35]. The improvement in the SF-36 physical function scale is in keeping with the improvement reported with fluticasone [11]. The diary card symptom scores changed in a comparable fashion to the health status measures. However, the lack of a validated symptom score for diary card data limits its quantitative interpretation. Nonetheless, significant changes were observed in the average diary card symptom score, which was the composite endpoint used to assess a range of COPD symptoms. Not every symptom was present in every individual, so the aggregate score tends to underestimate the benefit in subjects who were symptomatic. However, it provides important additional evidence that improvements in clinically relevant symptoms were occurring more frequently in subjects receiving ICS therapy.

The incidence and nature of adverse events were in keeping with those reported previously, with more subjects reporting symptoms of pharyngitis and a hoarse voice in the ICS group than in the placebo group. In the ICS group, the number needed to harm by causing a case of pneumonia was 49. Spontaneous bruising was a frequent finding in subjects receiving placebo but was more frequent with both ICS regimens. This might reflect greater bioavailability of MF, as a reduction in the plasma cortisol was observed in the subgroup of subjects in which this was recorded. However, these changes were modest, with values within the established normal range and no clinically significant hypoadrenalism identified. In the subgroup in which BMD measurements were made, there was evidence of some spontaneous improvement in the placebo group, a finding also noticed in the larger data set from the second Lung Health study [36]. This may reflect between-measurement variation in this test, and the apparent reduction in BMD with the MF-DPI BID regimen may also be a consequence of this variability. The lack of change in BMD seen with the MF-DPI QD regimen was encouraging and in keeping with the findings reported in the larger series of BMD measurements made in US participants in the TORCH study [26]. As in that report and in the INSPIRE study comparing tiotropium and the fluticasone propionate/salmeterol combination [37], we saw more episodes of pneumonia in the patients who received the mometasone treatment compared to those who did not. Like these other reports, treatment with an inhaled corticosteroid was associated with better health status and fewer exacerbations. The nature of these relatively infrequent events requires further clarification but large patient populations will be needed to achieve this.

## Conclusion

In summary, the current findings in subjects with moderate-to-severe COPD provide further evidence of an effect of ICS treatment on a number of clinically relevant endpoints and demonstrate that MF has benefits similar to those reported for other ICS therapies. The observation of greater improvements in lung function in ex-smokers requires further prospective testing, but does suggest that this group of patients may benefit with ICS therapy. Longer-term changes in lung function should be examined in this patient population. Finally, the results demonstrate that once-daily therapy with inhaled corticosteroids is as effective as dividing the dose into a morning and evening regimen in COPD patients. This has practical relevance as inhaled therapy producing once-daily bronchodilatation is now available, [38] and the benefits of using inhaled corticosteroids in COPD are greater when they are used as part of a combination regimen [26]. Our data suggest that MF can be used in a similar fashion to once-daily bronchodilator drugs, with the potential advantages of improved treatment adherence and convenience for the patient.

## Abbreviations

ATS: American Thoracic Society; BID: twice a day; BDP: beclomethasone dipropionate; BMD: bone mineral density; COPD: chronic obstructive pulmonary disease; DPI: dry powder inhaler; DXA: dual energy X-ray absorptiometry; FEF_25%–75%_: forced expiratory flow (L/s) between 25% and 75% of vital capacity; FEV_1_: forced expiratory volume (L) in one second; FVC: forced vital capacity (L); GOLD: Global Initiative for COPD; ICS: inhaled corticosteroid; MCS: mental health component summary of the SF-36; MF: mometasone furoate; MF-DPI: mometasone furoate delivered via a dry powder inhaler; PCS: physical health component summary of the SF-36; QD PM: once daily in the evening; SD: standard deviation; SF-36: short form 36; SGRQ: St. George's respiratory questionnaire.

## Competing interests

PMAC has spoken at an ERS evening symposium supported by the sponsors of this study and has conducted research into the role of other inhaled corticosteroids in COPD. SR has received research support for the study in the current manuscript; he has served as a consultant to Schering-Plough and as a data monitoring board for a separate Schering-Plough study; he has conducted clinical trials evaluating harm reduction products and has consulted with RJR Tobacco; he has consulted with and conducted clinical trials with several corporations with a general interest in COPD and inhaled glucocorticoids that may have an indirect benefit from the current study. AE has no competing interests. HSN has been a consultant, a member of the speakers' bureau, and a recipient of research grants from Schering-Plough Corporation. JPK has served as a consultant, is a member of the speakers' bureau, and has helped to develop educational materials for Schering-Plough Corporation. HS has been employed since 1998 as a VP of Allergy/Respiratory Clinical Research at Schering-Plough Corporation. As part of his compensation package he has received salary, bonuses, stock options and stock award.

## Authors' contributions

HS participated in the design of the study and was the responsible medical officer on the sponsor's clinical study report. PMAC, SR, HSN, EA, and JK were principal investigators in the study. PMAC drafted the manuscript and all authors read and approved the final manuscript.

## References

[B1] Mannino DM, Buist AS (2007). Global burden of COPD: risk factors, prevalence, and future trends. Lancet.

[B2] Chartbook on Cardiovascular, Lung, and Blood Diseases. http://www.nhlbi.nih.gov/resources/docs/cht-book.htm.

[B3] Hogg JC, Chu F, Utokaparch S, Woods R, Elliott WM, Buzatu L, Cherniack RM, Rogers RM, Sciurba FC, Coxson HO, Pare PD (2004). The nature of small-airway obstruction in chronic obstructive pulmonary disease. N Engl J Med.

[B4] Jeffery PK (1998). Structural and inflammatory changes in COPD: a comparison with asthma. Thorax.

[B5] Barnes PJ, Pedersen S, Busse WW (1998). Efficacy and safety of inhaled corticosteroids. New developments. Am J Respir Crit Care Med.

[B6] Hattotuwa KL, Gizycki MJ, Ansari TW, Jeffery PK, Barnes NC (2002). The effects of inhaled fluticasone on airway inflammation in chronic obstructive pulmonary disease: a double-blind, placebo-controlled biopsy study. Am J Respir Crit Care Med.

[B7] Sutherland ER, Allmers H, Ayas NT, Venn AJ, Martin RJ (2003). Inhaled corticosteroids reduce the progression of airflow limitation in chronic obstructive pulmonary disease: a meta-analysis. Thorax.

[B8] (2000). Effect of inhaled triamcinolone on the decline in pulmonary function in chronic obstructive pulmonary disease. N Engl J Med.

[B9] Burge PS, Calverley PM, Jones PW, Spencer S, Anderson JA, Maslen TK (2000). Randomised, double blind, placebo controlled study of fluticasone propionate in patients with moderate to severe chronic obstructive pulmonary disease: the ISOLDE trial. BMJ.

[B10] Paggiaro PL, Dahle R, Bakran I, Frith L, Hollingworth K, Efthimiou J (1998). Multicentre randomised placebo-controlled trial of inhaled fluticasone propionate in patients with chronic obstructive pulmonary disease. International COPD Study Group. Lancet.

[B11] Spencer S, Calverley PM, Sherwood Burge P, Jones PW (2001). Health status deterioration in patients with chronic obstructive pulmonary disease. Am J Respir Crit Care Med.

[B12] Valk P van der, Monninkhof E, Palen J van der, Zielhuis G, van Herwaarden C (2002). Effect of discontinuation of inhaled corticosteroids in patients with chronic obstructive pulmonary disease: the COPE study. Am J Respir Crit Care Med.

[B13] (2006). Global strategy for the diagnosis, management, and prevention of chronic obstructive pulmonary disease. http://www.goldcopd.com/Guidelineitem.asp?l1=2&l2=1&intId=989.

[B14] Rabe KF, Hurd S, Anzueto A, Barnes PJ, Buist SA, Calverley P, Fukuchi Y, Jenkins C, Rodriguez-Roisin R, van Weel C, Zielinski J (2007). Global Strategy for the Diagnosis, Management, and Prevention of Chronic Obstructive Pulmonary Disease: GOLD Executive Summary. Am J Respir Crit Care Med.

[B15] (2004). Chronic obstructive pulmonary disease. National clinical guideline on management of chronic obstructive pulmonary disease in adults in primary and secondary care. Thorax.

[B16] Nayak AS, Banov C, Corren J, Feinstein BK, Floreani A, Friedman BF, Goldsobel A, Gottschlich GM, Hannaway PJ, Lampl KL, Lapidus RJ, Lawrence M, Lumry W, Munk Z, Pearlman D, Scardella AT, Schenkel EJ, Segal AT, Segall N, Silverman B, Shneyer L, Nolop KB, Harrison JE (2000). Once-daily mometasone furoate dry powder inhaler in the treatment of patients with persistent asthma. Ann Allergy Asthma Immunol.

[B17] Noonan M, Karpel JP, Bensch GW, Ramsdell JW, Webb DR, Nolop KB, Lutsky BN (2001). Comparison of once-daily to twice-daily treatment with mometasone furoate dry powder inhaler. Ann Allergy Asthma Immunol.

[B18] D'Urzo A, Karpel JP, Busse WW, Boulet LP, Monahan ME, Lutsky B, Staudinger H (2005). Efficacy and safety of mometasone furoate administered once-daily in the evening in patients with persistent asthma dependent on inhaled corticosteroids. Curr Med Res Opin.

[B19] Karpel JP, Busse WW, Noonan MJ, Monahan ME, Lutsky B, Staudinger H (2005). Effects of mometasone furoate given once daily in the evening on lung function and symptom control in persistent asthma. Ann Pharmacother.

[B20] Fish JE, Karpel JP, Craig TJ, Bensch GW, Noonan M, Webb DR, Silverman B, Schenkel EJ, Rooklin AR, Ramsdell JW, Nathan R, Leflein JG, Grossman J, Graft DF, Gower RG, Garay SM, Frigas E, Degraff AC, Bronsky EA, Bernstein DI, Berger W, Shneyer L, Nolop KB, Harrison JE (2000). Inhaled mometasone furoate reduces oral prednisone requirements while improving respiratory function and health-related quality of life in patients with severe persistent asthma. J Allergy Clin Immunol.

[B21] (1995). Standardization of Spirometry, 1994 Update. American Thoracic Society. Am J Respir Crit Care Med.

[B22] Crapo RO (1989). Reference values for lung function tests. Respir Care.

[B23] Calverley P, Pauwels R, Vestbo J, Jones P, Pride N, Gulsvik A, Anderson J, Maden C (2003). Combined salmeterol and fluticasone in the treatment of chronic obstructive pulmonary disease: a randomised controlled trial. Lancet.

[B24] Calverley PM, Boonsawat W, Cseke Z, Zhong N, Peterson S, Olsson H (2003). Maintenance therapy with budesonide and formoterol in chronic obstructive pulmonary disease. Eur Respir J.

[B25] Pauwels RA, Lofdahl CG, Laitinen LA, Schouten JP, Postma DS, Pride NB, Ohlsson SV (1999). Long-term treatment with inhaled budesonide in persons with mild chronic obstructive pulmonary disease who continue smoking. European Respiratory Society Study on Chronic Obstructive Pulmonary Disease. N Engl J Med.

[B26] Calverley PM, Anderson JA, Celli B, Ferguson GT, Jenkins C, Jones PW, Yates JC, Vestbo J (2007). Salmeterol and fluticasone propionate and survival in chronic obstructive pulmonary disease. N Engl J Med.

[B27] Scanlon PD, Connett JE, Waller LA, Altose MD, Bailey WC, Buist AS (2000). Smoking cessation and lung function in mild-to-moderate chronic obstructive pulmonary disease. The Lung Health Study. Am J Respir Crit Care Med.

[B28] Burge PS, Calverley PM, Jones PW, Spencer S, Anderson JA (2003). Prednisolone response in patients with chronic obstructive pulmonary disease: results from the ISOLDE study. Thorax.

[B29] Chaudhuri R, Livingston E, McMahon AD, Thomson L, Borland W, Thomson NC (2003). Cigarette smoking impairs the therapeutic response to oral corticosteroids in chronic asthma. Am J Respir Crit Care Med.

[B30] Ito K, Ito M, Elliott WM, Cosio B, Caramori G, Kon OM, Barczyk A, Hayashi S, Adcock IM, Hogg JC, Barnes PJ (2005). Decreased histone deacetylase activity in chronic obstructive pulmonary disease. N Engl J Med.

[B31] Jones PW, Willits LR, Burge PS, Calverley PM (2003). Disease severity and the effect of fluticasone propionate on chronic obstructive pulmonary disease exacerbations. Eur Respir J.

[B32] Szafranski W, Cukier A, Ramirez A, Menga G, Sansores R, Nahabedian S, Peterson S, Olsson H (2003). Efficacy and safety of budesonide/formoterol in the management of chronic obstructive pulmonary disease. Eur Respir J.

[B33] Calverley PM, Spencer S, Willits L, Burge PS, Jones PW (2003). Withdrawal from treatment as an outcome in the ISOLDE study of COPD. Chest.

[B34] Spencer S, Calverley PM, Burge PS, Jones PW (2004). Impact of preventing exacerbations on deterioration of health status in COPD. Eur Respir J.

[B35] Wouters EF, Postma DS, Fokkens B, Hop WC, Prins J, Kuipers AF, Pasma HR, Hensing CA, Creutzberg EC (2005). Withdrawal of fluticasone propionate from combined salmeterol/fluticasone treatment in patients with COPD causes immediate and sustained disease deterioration: a randomised controlled trial. Thorax.

[B36] Scanlon PD, Connett JE, Wise RA, Tashkin DP, Madhok T, Skeans M, Carpenter PC, Bailey WC, Buist AS, Eichenhorn M, Kanner RE, Weinmann G (2004). Loss of bone density with inhaled triamcinolone in Lung Health Study II. Am J Respir Crit Care Med.

[B37] Wedzicha JA, Calverley PM, Seemungal TA, Hagan G, Ansari Z, Stockley RA (2008). The prevention of chronic obstructive pulmonary disease exacerbations by salmeterol/fluticasone propionate or tiotropium bromide. Am J Respir Crit Care Med.

[B38] Calverley PM, Lee A, Towse L, van Noord J, Witek TJ, Kelsen S (2003). Effect of tiotropium bromide on circadian variation in airflow limitation in chronic obstructive pulmonary disease. Thorax.

